# Letter to the Editor: About the quality and impact of scientific articles

**DOI:** 10.1007/s11192-017-2374-2

**Published:** 2017-04-10

**Authors:** Martin Ricker

**Affiliations:** Instituto de Biología, Universidad Nacional Autónoma de México (UNAM), Circuito Zona Deportiva, Ciudad Universitaria, 04510 Ciudad de México, Mexico

**Keywords:** Article impact, Article quality, Citation analysis, Peer review

## Abstract

It is argued that counting the total number of times a scientific article is cited by others, does neither result in a proxy for its cognitive impact nor for its quality. One would have to distinguish at least substitutable and fundamental references. A supposed correlation between peer review assessments and citation counts is conceptually problematic, because peer review includes objective as well as subjective considerations (convictions). With refined methods, however, a differential citation analysis might be able in the future to answer if a given article did or did not have positive cognitive impact on subsequent research.

Bornmann and Haunschild (2017) argue that by broadening the meaning of the term “impact” on societal impact, scientometrics is likely to lose its focus on quality, where “quality of publications was measured as a rule in terms of the number of citations” (p. 938). Furthermore they state that “impact of research might no longer be seen as a proxy for its quality” (p. 939). I like to comment on their assumption that

1 $${\text{citation count for article}} \approx {\text{its impact}} \approx {\text{its quality}}.$$

Scientometrics, bibliometrics, informetrics, etc. emphasize their focus on mathematical and statistical analyses (see Milojević and Sugimoto 2012; Zhang et al. 2013). Statistics helps to analyze data logically, and to reveal patterns and conclusions that may not be immediately obvious, but ultimately you can always express in plain English what information is provided by the original data. Does citation count data contain the information about cognitive impact and article quality?

It is widely acknowledged that there are different motives for scientists to cite one another (Nicolaisen 2007). For example, Camacho-Miñano and Núñez-Nickel (2009: 757) distinguish nine categories, while Ricker (2015: 208) proposed to distinguish at least “substitutable citations” (called “perfunctory” in Waltman et al. 2013: 636) and “fundamental citations” (with cognitive impact). Substitutional citations are almost randomly chosen from a pool of thematically related articles, just to show that others have worked in the field. Most citations are substitutable (Alves Ramos et al. 2012; Ricker 2015: 208; Simkin and Roychowdhury 2005). Fundamental citations are the real target of evaluative citation analysis. If one lumps together a majority of substitutable citations and a remainder of fundamental citations in the data, one does not get an indicator of cognitive impact. The conclusion that citation counts are a proxy for cognitive impact becomes logically wrong. I am not aware of any evaluative scientometric analysis up to date, where the distinction has been made.

The term “quality” is even more difficult to connect with citation counts. To judge quality, one has to define the intrinsic characteristics that define it. In scientific articles, generally considered key characteristics are a significant contribution to current knowledge and expansion of the scientific frontier, creativity, clarity of exposition, and possibly applications. Time and again, the severe problem for the scientific process of using citation counts of scientists’ articles as quality and impact parameters for evaluating the authors, has been pointed out (e.g., Adler and Harzing 2009; Allen et al. 2009; Belcher et al. 2016; Chavalarias 2017; De Bellis 2009: chapter 7; Gagolewski 2013; Hicks et al. 2015; Kaur et al. 2015; Ricker et al. 2009, 2010). Bornmann and Haunschild (2017) are aware of the problem, as they cite a number of studies (e.g., MacRoberts and MacRoberts 2010), and discuss the issue, also in previous own work (e.g., Bornmann et al. 2008). Their justification to go ahead with (1) is a supposed correlation between citation counts and peer review judgment (p. 938). To assume such a correlation is conceptually problematic, and would be the topic of a critical review by itself. Aksnes and Taxt (2004: 39) point out that a positive correlation between peer judgements and bibliometric performance measures can only be expected if the aspects assessed by the peers correspond to those reflected through bibliometric indicators. This, however, makes the argument largely circular. Furthermore, peers use generally a threshold approach to evaluation, after weighting all criteria: accept versus reject a manuscript, or interesting versus irrelevant publication for a given purpose. They rarely think about assigning a score on an open scale, like it is the case with citation counts.

Finally, and more fundamentally, peer evaluators make an informed, but partially subjective assessment, even when attempting to be as fair as possible. A justification is objective if in principle it can be tested and understood by anybody, while subjectivity refers to feelings of conviction (Popper 1959: 44, citing Immanuel Kant). Expert evaluations include objective and subjective elements. An “objective *evaluation*” is impossible, because *values* are subjective. Only an evaluation taking into account objective facts, such as scientometric indicators, is possible. The idea to automate evaluation completely with an objective algorithm, that has been discussed sometimes in scientometrics (e.g., Good et al. 2015; Nicolini et al. 1995), is as impossible as substituting a panel of supreme court judges with a computer. If one tells a computer to find the research applicant with the highest number of citations, then your subjective preference is to maximize the number of citations. One could have different preferences, such as finding a researcher who works with particular methods, or who works on the best applications for society (Hicks et al. 2015; Sutherland et al. 2011). Bibliometric indicators unfortunately often become “pseudo-objective”, when decision-makers use them as automated evaluation tools, with the necessary and unavoidable preference statements hidden behind them. The concept of preference statements of expert panels (see Ricker 2015), as implemented for the interface of producers and consumers in economics (and also econometrics), is notoriously missing in scientometrics!

One can come up with examples, where the goal of publishing a high-quality article is not related with a supposed goal to get widely cited. In my own institute, an important activity is the description of plant and animal species that are new to science. Assume that a new tree species is published in a taxonomic journal. Furthermore, assume that the article will receive in the future (maybe decades later) a single citation as a recognized species in a taxonomic revision. Then the mission of the original publication has been completed, with a single citation! It is all the cognitive impact you need to justify the publication, and the argument that it is a low-quality publication is wrong.

A good reflection for employing statistical models was formulated by the statistician George E. P. Box (1919–2013): “All [here scientometric] models are wrong, but some are useful” (Box et al. 2005: 384). Given the popularity that scientometrics and related fields have gained nowadays for assisting and often *de facto* replacing traditional research evaluation (e.g., Butler 2008; Good et al. 2015; Ricker 2015), scientists working in the field have also gained a lot of responsibility. Fortunately, there are some interesting developments in scientometrics, coming along with ever more advanced possibilities to analyze scientific texts with computers. First, it has been recognized that citation analysis can be carried out in much more detail: Which references are cited only once, and which ones more than once in the same text? One expects that recurrence of citations in a single article has to do with a higher importance for the citing author. In which sections are references cited? In the introduction, in the methods, or in the discussion? (see, e.g., Ding et al. 2013; Hu et al. 2015). The introduction tends to include substitutable citations, while citations in the methods or discussion sections are expected to indicate a more transcendental role, related to cognitive impact. Second, scientific text can be analyzed semantically in ever more detail: Which text segments have appeared in previous articles in similar form? How distant are semantically articles of the same authors from each other? How distant is a given article from other articles in the same scientific field? In which semantic context is an article cited? (see, e.g., Bertin et al. 2016; Gerrish and Blei 2010; Milojević 2015).

Easier than to quantify the impact of an article on a numerical scale would be to tackle the question if a given article did or did not have positive cognitive impact on subsequent research. With refined methods in the future, possibly a differential citation analysis will be able to answer this question automatically. Such a development I would call a “revolution in scientometrics”, sensu Bornmann and Haunschild (2017).
